# *SlSPL15* : A Negative Regulator Targeted by SlmiR156a Participates in Regulating the Light‐Induced Anthocyanin Biosynthesis of *Aft* Tomato Fruits

**DOI:** 10.1111/ppl.70471

**Published:** 2025-08-30

**Authors:** Chuyao Xu, Siyue Qi, Fuchang Guo, Hui Wang, Ji Li, Jiazhen Li, Weilin Wu, Bo Zhou

**Affiliations:** ^1^ College of Life Science Northeast Forestry University Harbin China; ^2^ Agricultural College Yanbian University Yanji China

**Keywords:** anthocyanin biosynthesis, light, miR156a, *Solanum lycopersicum*, SPL/SBPs, tomato

## Abstract

Anthocyanins are crucial compounds known for their antioxidant and health benefits. The *Aft* tomato ( *Solanum lycopersicum* ) line LA1996 can generate anthocyanins in fruits upon light exposure. Despite the identification of various regulatory genes, such as microRNAs and transcription factors involved in anthocyanin biosynthesis across different plant species, the function of the miR156/SPL module in *Aft* tomato fruit pigmentation is not well understood. In this research, 17 *SlSPL* family genes of *Aft* tomato were classified into six clades. *SlSPL15* (Solyc10T002263.1) was grouped in Clade V, with *AtSPL9*, which is known to be involved in anthocyanin biosynthesis in *Arabidopsis*. Moreover, an inverse relationship between *SlSPL15* and miR156a expression in mature green (MG) stage fruits was shown by quantitative RT‐PCR analysis. Transient expression analysis of luciferase confirmed that *SlSPL15* transcripts are degraded by SlmiR156a. Furthermore, overexpression of *SlSPL15* in *Aft* tomato reduced the anthocyanin accumulation in MG fruits. In *SlSPL15* overexpressed tomato plants, the transcription level of *SlSPL15* was elevated compared to that in wild‐type fruits, whereas the expression of *SlPAL*, *SlCHS1*, *SlDFR*, *SlF3H*, *SlF3′5′H*, and *SlANS* was reduced. Additionally, the expression of light‐responsive regulatory genes *SlHY5*, *SlAN2‐like*, and *SlMYB12* in the anthocyanin biosynthetic pathway was also reduced in light‐exposed fruits of *35S:SlSPL15* plants. Subcellular localization analysis verified that SlSPL15 is localized in the nucleus, while yeast two‐hybrid assays demonstrated its interaction with *SlAN2‐like*, a part of the MBW complex that participates in regulating anthocyanin biosynthesis in *Aft* tomato fruits. The findings expand our comprehension of the roles of *SlSPL15*, targeted by SlmiR156a, in regulating light‐induced anthocyanin accumulation in tomatoes.

## 1 Introduction

Tomato ( *Solanum lycopersicum* ) is widely valued for its antioxidant properties, primarily due to its high content of lycopene and flavonoids. However, anthocyanins, which contribute to the purple pigmentation in fruits, are typically absent in most cultivated tomato varieties, except for those carrying the *Aft* (Anthocyanin fruit) gene. The LA1996 tomato genotype, which harbors the dominant *Aft* locus, exhibits a unique light‐responsive synthesis of anthocyanins in the fruit epidermis (Sun et al. 2020). Light acts as a pivotal environmental cue, modulating various metabolic pathways, including the biosynthesis of anthocyanins (Ma, Ma, et al. 2021). Recent studies indicate that the transcription factors MYB, bHLH, and WDR are essential components of the MBW complex, which directly interacts with gene promoters in the anthocyanin biosynthetic pathway to regulate anthocyanin production (Broun 2005; S. Li 2014; P. Xu et al. 2021; W. Xu et al. 2015). Additionally, light‐induced anthocyanin biosynthesis is mediated through the HY5‐dependent pathway, which further activates the MBW complex, highlighting its role in this light‐dependent regulatory process (Bai et al. 2019; Ma, Ma, et al. 2021).

SPL (SQUAMOSA promoter‐binding protein‐like) transcription factors, characterized by their conserved SBP domain, are vital regulators of plant growth and development (Cai et al. 2018; Ferreira e Silva et al. 2014). Recent research suggests that miR156 modulates plant growth and development through the suppression of specific members of the *SPL* gene family (Dong et al. 2021; Ferreira e Silva et al. 2014; Guo et al. 2017; Wang et al. 2009). SPL transcription factors can bind to sites with a GTAC core motif, enabling them to regulate the expression of specific target genes (Yao et al. 2019). In *Arabidopsis*, miR156 targets 10 out of 16 *SPL* genes (Gandikota et al. 2007; Wu and Poethig 2006), while in rice, it regulates 11 of the 19 *SPL* genes (Xie et al. 2006). In tomatoes, *SPL* family members have been identified to contain potential binding sites for both miR156 and miR157 (Salinas et al. 2012). The transcription factor AtSPL9, regulated by miR156, competes with bHLH proteins to form a complex with PAP1, which negatively affects anthocyanin accumulation in the inflorescent stem of *Arabidopsis* (Gou et al. 2011). Additionally, AtSPL3/4/5 interact with FD (a bZIP transcription factor) to mediate MADS‐box genes during flower development (Jung et al. 2016). In rice, multiple *OsSPL* genes exhibit functional redundancy and antagonistically regulate the development of vegetative and inflorescence meristems (Liu et al. 2016). For instance, *OsSPL17* is redundant with *OsSPL14* in regulating rice panicle branching (Jiao et al. 2010; Wang et al. 2015). In blueberries, miR156a‐targeted *VcSPL12* interacts with VcMYBPA1 to repress genes in the anthocyanin biosynthetic pathway (Li et al. 2020). Furthermore, in poplar, miR156 overexpression decreases the transcription levels of *SPL15*, *SPL17*, and *SPL24*, leading to increased anthocyanin accumulation (Wang et al. 2020). The PyPIF5‐PymiR156a‐PySPL9‐PyMYB114/MYB10 regulatory module mediates light‐dependent anthocyanin biosynthesis in red pears (Liu et al. 2021). In apple skin, the lncRNAs MLNC3.2 and MLNC4.6 act as endogenous target mimics of miR156a, inhibiting its cleavage of *SPL2‐like* and *SPL33* mRNA during light‐mediated anthocyanin biosynthesis (Yang et al. 2019). In tomatoes, miR156a‐targeted *SlSPL13* has been shown to regulate inflorescence structure and lateral branch production (Chen et al. 2023; Cui et al. 2020). Moreover, miR156‐targeted *SlSBP* genes, such as *SlSBP3* and *SlSBP15*, are crucial for regulating floral meristem determinacy and ovary patterning (Ferigolo et al. 2023). SlSBP15 has also been reported to inhibit shoot branching in tomato by regulating auxin transport and forming protein complexes with GOBLET and BRANCHED1b (Barrera‐Rojas et al. 2023). Furthermore, SlymiR157‐targeted LeSPL‐CNR influences tomato fruit ripening (Chen et al. 2015). Although previous studies have deciphered the roles of certain *SPL* genes in tomato development, the functional diversity of the *SPL* gene family still requires further exploration.

Recent studies have uncovered various miRNAs and transcription factors that regulate both anthocyanin biosynthesis and pathways related to plant growth and development (Castillejo et al. 2020; Karlova et al. 2013; Qian et al. 2017; Zhou et al. 2020). However, the specific role of *SPLs* targeted by miR156 in light‐induced fruit pigmentation in tomatoes has not been fully clarified. In our research, we found that miR156a targets SlSPL15, which interacts with SlAN2‐like, and *SlSPL15* overexpression negatively affects light‐induced anthocyanin accumulation in the fruits of *Aft* tomatoes. This suggests a complex regulatory mechanism in which the miR156a‐SlSPL15 module plays multifaceted roles in plant physiology, particularly in fruit pigmentation in tomatoes. These results not only enhance our understanding of miRNA‐mediated regulation in plant pigmentation but also pave the way for future research to explore the environmental factors influencing this regulatory pathway.

## 2 Materials and Methods

### 2.1 Plant Materials and Light Treatments

Tomato cultivar LA1996 ( *S. lycopersicum* ) seeds were acquired from the University of California, Davis Tomato Genetic Resource Center (http://tgrc.ucdavis.edu/). These seeds were cultivated in 20‐cm pots using locally sourced soil from Northeast China (which is also known as black soil) within a greenhouse at Northeast Forestry University in Harbin (126°37′E, 45°42′N), China. Upon reaching the mature green (MG) developmental stage, the tomato fruits were harvested for shading treatment. Each fruit was divided, with one half exposed to LED light, while the other half was shielded with aluminum foil. The treatment was performed at 22°C under a 16 h light/8 h dark photoperiod using T5 LED (Zhejiang Yangguang Lighting), at a light intensity of approximately 150 W m^−2^ and relative humidity maintained at 65%. After 7 days of LED light exposure, the fruits were collected as samples for RNA isolation and anthocyanin analysis.

### 2.2 Measurement of Anthocyanin Content

A fruit peel sample, approximately 0.1 g or 1 cm^2^ of epidermis from either the light or shading treatment, was ground and homogenized in liquid nitrogen. Pigment extraction was performed as previously described (Povero et al. 2011), with acidified methanol (1% HCl, v/v) by continuous shaking overnight at 4°C. After extraction, samples were centrifuged at 13,800*g* for 2 min. Following extraction, the anthocyanin content was quantified by calculating the ratio of (*A*_530_ − 0.25 * *A*_657_) to the gram of fresh mass. Results were expressed with vertical bars representing ± standard error (SE) for three replicates (*n* = 3). Statistical analyses employed Student's t‐tests, considering a *p*‐value below 0.05 as statistically significant.

### 2.3 Analysis of Phylogenetic Relationships Among SPL Proteins

The SlSPL sequences of tomato were sourced from Phytozome (https://phytozome‐next.jgi.doe.gov/), while the AtSPL protein sequences of *Arabidopsis* were retrieved from the PlnTFDB database (http://planttfdb.gao‐lab.org/download.php). SPL protein sequences from tomato and *Arabidopsis* were aligned using Clustal Omega (https://www.ebi.ac.uk/jdispatcher/msa/clustalo). The alignment results were used to construct a neighbor‐joining phylogenetic tree in MEGA7.0, employing 1000 bootstrap replicates for reliability (Kumar et al. 2016).

### 2.4 Synteny and Physical Location Assessment

The chromosomal mapping of *SlSPL* genes was carried out using the TBtools software (Chen, Wu, et al. 2023). Collinearity analysis was performed with MCScanX to identify tandem and fragment repeat events within the tomato genome (Wang et al. 2012). To further investigate the collinearity relationships, a mapping of evolutionary relationships among paralogous and orthologous gene pairs was conducted, and a fragment repeat map was created using the Circos online mapping tool (Chen et al. 2022). Furthermore, the impact of selection pressure on the protein‐coding genes of SlSPLs was evaluated by calculating the Ka/Ks substitution rate for paralogous SlSPL gene pairs with TBtools (Chen, Wu, et al. 2023).

### 2.5 Motif and MRE Analysis of the SlSPL Family Genes

SBP motifs were identified using InterPro (https://www.ebi.ac.uk/interpro/) and the MEME program (http://meme‐suite.org/tools/meme) with default settings (Bailey et al. 2009). The WebLogo3 platform (http://weblogo.berkeley.edu/logo.cgi) was utilized to generate a sequence logo for the SlSBP domain in tomato (Crooks et al. 2004). Additionally, the prediction of miR156 targeted sites (MRE) was carried out using the psRNATarget tool (https://www.zhaolab.org/psRNATarget/) (Dai et al. 2018).

### 2.6 MIR156a Effector Vector and SlSPL15 MRE Reporter Vector Construction

The polymerase chain reaction (PCR) product of the SlMIR156a precursor fragment was digested with *Xho*I and *BamH*I and subsequently ligated into the pA7‐GFP vector. The recombinant plasmid DNA was then digested with *Hind*III and *EcoR*I before being ligated to the digested pCAMBIA‐1300 vector to obtain the effector vector. For the reporter vector, a *T7* promoter was inserted upstream of the *LUC* gene in the pGREENII‐0800 vector using the *pT7‐LUC* primer for PCR and Exnase II (ClonExpress II One Step Cloning kit from Vazyme). The modified plasmid DNA was then used as a template for overlapping PCR to insert the sequence of the MRE site (the binding sites for SlmiR156a and *SlSPL15*) into the 3′ untranslated region of *LUC*, resulting in the construction of the expression vector.

### 2.7 Transient Expression Analysis of *LUC* to Identify the Cleavage of SlmiR156a to *SlSPL15*

The constructs *pGreenII0800‐pT7:LUC‐MRE* and *pCAMBIA1300‐p35S:preMIR156a* were transformed into the *A. tumefaciens* GV3101 strain. Prior to infection, the *Agrobacterium* containing the target genes was cultured for 2 days in liquid YEB medium supplemented with the appropriate antibiotics. Then bacterial cultures were activated in YEB medium with acetosyringone (200 μM) and MES (10 mM) for 24 h, followed by centrifugation at 4000*g*. The resuspended cells in MMA medium (OD₆₀₀ = 0.6) were infiltrated into the leaves of *N. benthamiana*, and luciferase signals were detected using a Tanon 4600SF system (Tanon Science & Technology Co. Ltd.) as described previously (Wang et al. 2022). Each combination of effector and reporter was tested in at least three replicates, yielding consistent results.

### 2.8 Vector Construction and Tomato Transformation

Total RNA was extracted from the epidermis of MG fruits of *Aft* tomato. The coding sequence fragment of *SlSPL15* (1132 bp) was amplified by real‐time PCR (RT‐PCR) and subsequently cloned into the pBI121 vector using *Bam*HI via the EasyGeno Assembly Cloning Kit (TIANGEN Biotechnology Co. Ltd.). Following sequencing to confirm the correct insertion, the recombinant plasmid was introduced into the *Agrobacterium* strain GV3101. Tomato transformation was subsequently performed using the *Agrobacterium tumefaciens* –mediated method, as detailed by Park et al. (2003). Kanamycin was used for selection to generate putative transgenic tomato plants overexpressing *SlSPL15*.

### 2.9 RNA Extraction and qRT‐PCR Analysis

The epidermis of MG stage fruits of *Aft*‐WT plants and OE‐SPL15 plants exposed to light or shading was harvested for RNA isolation (refer to the TRNzol Universal Reagent instructions from Tiangen Biotech Co. Ltd.). cDNA synthesis and quantitative RT‐PCR (qRT‐PCR) analyses were conducted using standardized protocols (Zhou et al. 2020). The primer sequences are detailed in Table S1. Each sample underwent triplicate analyses, with the *Actin* gene serving as an internal control for normalization. Transcript levels were quantified using the comparative CT (∆∆CT) (Livak and Schmittgen 2001) or (∆CT) (Schmittgen and Livak 2008) method. Statistical analysis was performed using Student's *t*‐tests, with significance determined at *p* < 0.05 across three biological replicates.

### 2.10 The Subcellular Localization of SlSPL15

The SlSPL15 coding sequence was cloned from a fruit cDNA library and linked to the C‐terminus of a GFP tag, creating the SlSPL15‐GFP construct within the pA7‐GFP vector at *SalI* and *SpeI* restriction sites. This recombinant plasmid, pA7‐SlSPL15‐GFP, was transiently expressed in onion epidermal cells through vacuum infiltration, following the protocol outlined by Zhou et al. (2009). After a 48‐h incubation, the infiltrated onion epidermis was mounted on slides and analyzed using a Zeiss LSM 510 META fluorescence microscope using a standard filter set. An empty GFP vector served as a control. For transient expression localization analysis in tobacco leaves, the *Agrobacterium* strain GV3101, containing the construct *35S:SlSPL15* in the pBI121‐GFP vector, was infiltrated into tobacco leaves. The construct *35S:GFP* was used as a control. After 72 h, GFP was detected using a Zeiss LSM 510 META fluorescence microscope (Wang, Song, et al. 2021).

### 2.11 Yeast Two‐Hybridization Analysis of the Interaction of SlSPL15 and SlAN2‐Like Proteins

The complete SlSPL15 and SlJAF13 sequences were amplified and inserted into the pBD‐GAL4 Cam vector. The *SlAN2‐like* gene was cloned into the pAD‐GAL4‐2.1 vector to investigate its interaction with SlSPL15 and SlJAF13. The recombinant vectors were verified for correct open reading frames through sequencing before being transformed into the yeast strain Y187. To assess autoactivation, SlSPL15‐BD transformed colonies were cultured on tryptophan‐deficient synthetic defined (SD) medium containing 5‐bromo‐4‐chloro‐3‐indolyl α‐d‐galactopyranoside (X‐α‐Gal). Colonies co‐transformed with both constructs were selected on SD medium deficient in leucine and tryptophan (double dropout medium) with X‐α‐Gal to verify protein interactions.

## 3 Results

### 3.1 Genomic Localization Analysis of the *SlSPL* Gene Family in Tomato ( *S. lycopersicum* ) and SlmiR156a Target Site Prediction?

**FIGURE 1:**
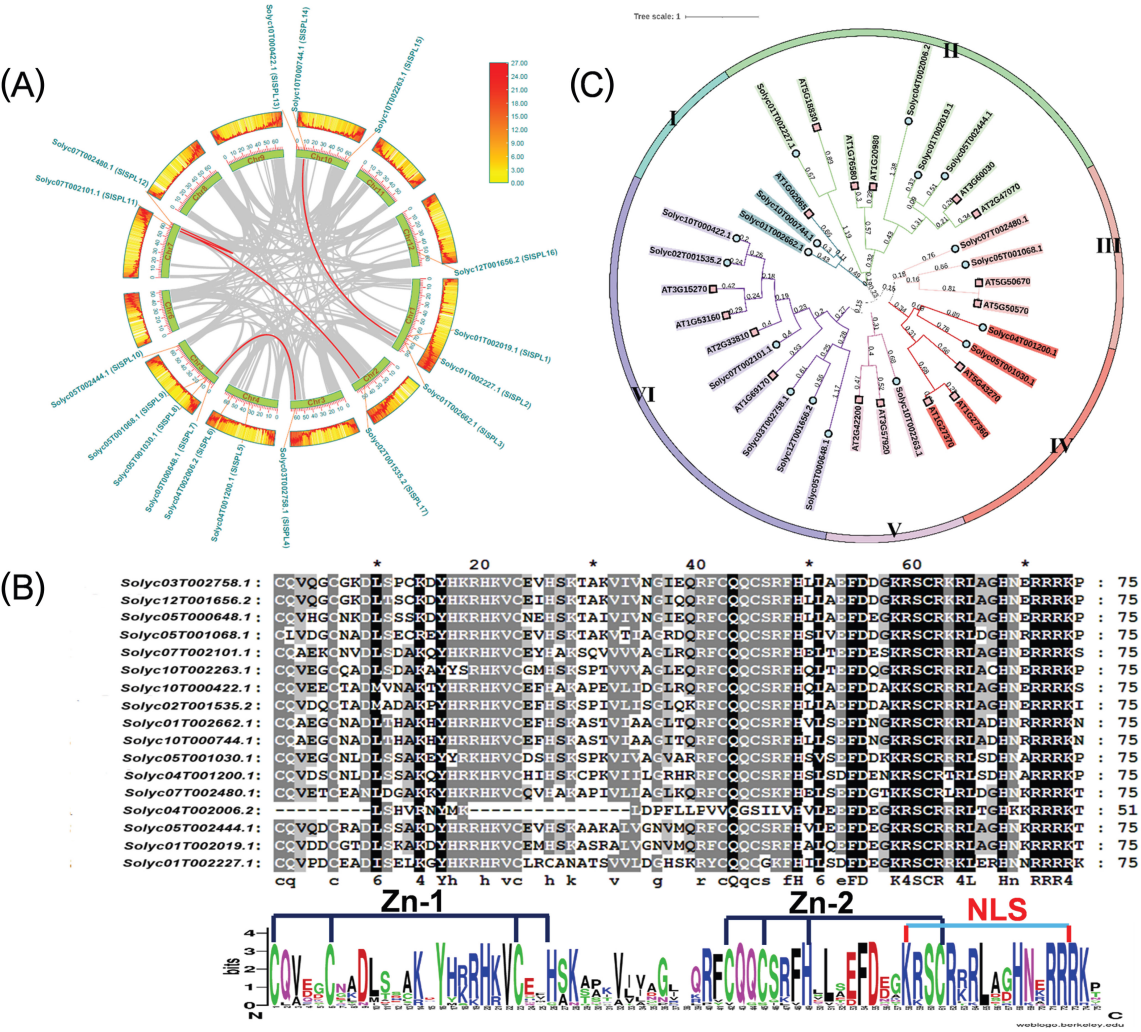
Collinearity, conserved domains and phylogenetic tree analysis in SlSPLs. (A) Chromosomal mapping and synteny analysis of *SlSPLs*. The gradient‐colored rectangles, arranged from the innermost to the outermost part, represent Chromosomes 1–12, with a heat map on the outermost track showing gene density. Gray lines at the center, represent synteny blocks within the tomato genome, and red lines connecting chromosomes denote segmental duplication gene pairs. (B) Sequence alignment of the conserved SBP motifs, which encompass a nuclear localization signal (NLS) and two zinc finger motifs (Zn‐1/2). (C) Phylogenetic analysis of SPLs in tomato and *Arabidopsis* based on amino acid sequences. The 34 *SPL* genes were classified into six groups. ■ represents the SPL genes in tomato; ● denotes the SPL genes in *Arabidopsis*.

The conserved SBP domain of *Arabidopsis thaliana* SPL proteins was used as a query in BLASTP searches against the *S. lycopersicum* genome (SL4.0 assembly) in Phytozome, yielding 17 putative *SlSPL* genes with full‐length SBP domains (*SlSPL1* to *SlSPL17*) distributed across eight chromosomes. MCScanX synteny analysis identified four gene pairs with segmental duplication across six chromosomes, highlighting the importance of segmental duplication in the genomic expansion of the SPL gene family in tomatoes (Figure 1A). Analysis of gene selection pressure revealed a Ka/Ks ratio < 1 for paralogous *SPL* gene pairs, suggesting strong purifying selection during evolution (Table S2). Multiple sequence alignment validated the presence of a conserved SBP domain in SlSPL proteins, which includes zinc finger motifs and a monopartite nuclear localization signal (Figure 1B). Target prediction analyses using psRNATarget (https://www.zhaolab.org/psRNATarget/) highlighted that several *SlSPLs*, including *SlSPL5* (Solyc04T001200.1), *SlSPL7* (Solyc05T000648.1), *SlSPL8* (Solyc05T001030.1), *SlSPL9* (Solyc05T001068.1), *SlSPL11* (Solyc07T002101.1), *SlSPL12* (Solyc07T002480.1), *SlSPL13* (Solyc10T000422.1), *SlSPL14* (Solyc03T002758.1), *SlSPL15* (Solyc10T002263.1), *SlSPL16* (Solyc12T001656.2), and *SlSPL17* (Solyc02T001535.2) were targeted by miR156a (Table S3). Phylogenetic tree construction of SPL orthologs from tomato and *Arabidopsis* classified 34 *SPL* genes into six groups (Groups I–VI). Notably, *SlSPL15* (Solyc10T002263.1) clustered with *AtSPL15* (AT3G57920.1) and *AtSPL9* (AT2G4200.1) in Group V, suggesting a similar function for *SlSPL15* in tomato as observed with *AtSPL15* and *AtSPL9* in *Arabidopsis* (Figure 1C). Consequently, we focused on exploring the role of *SlSPL15* in anthocyanin synthesis in *Aft* tomatoes.

### 3.2 The Accumulation of Anthocyanin in *Aft* Tomato Fruits Is Light‐Dependent and Is Associated With the Transcription Level of the SlmiR156a/ *SlSPL15* Module

**FIGURE 2:**
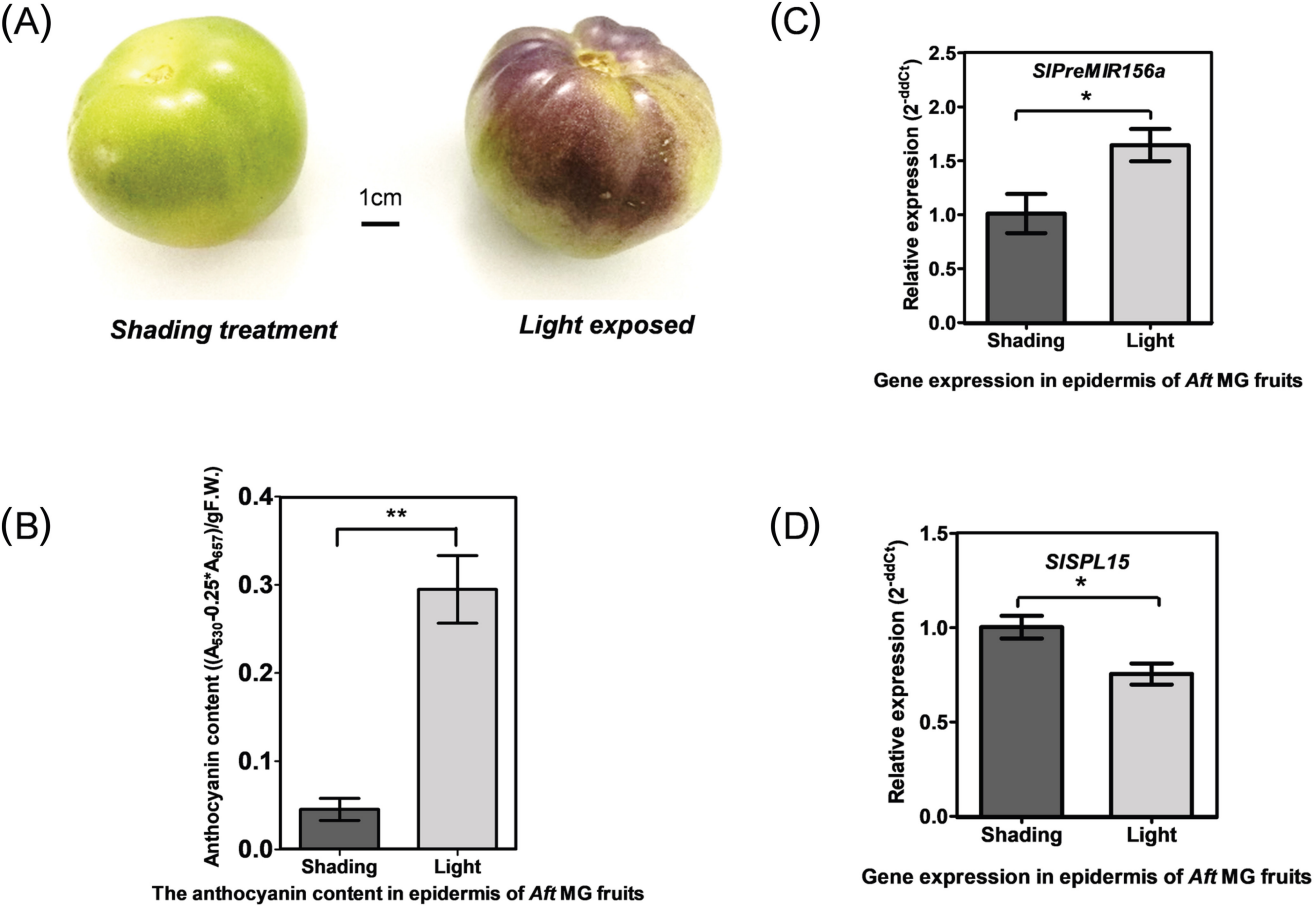
Light‐responsive anthocyanin accumulation and the expression patterns of *SlPreMIR156a* and *SlSPL15* in mature green (MG) development stage fruits of *Aft* tomatoes. (A) Light‐induced anthocyanin biosynthesis of tomato (LA1996) fruits with light exposure and shading treatment. (B) Anthocyanins were extracted from the peels of fruits subjected to light and shading treatment, and the concentration was measured using the formula (*A*_530_ − 0.25 * *A*_657_) per gram of fresh mass. Error bars represent ± standard error (SE) (*n* = 3). (C) The expression of *SlPreMIR156a* in shading and light‐exposed treatment of fruits. (D) The expression of *SlSPL15* in shading and light‐exposed treatment of fruits. Each bar shows the mean ± SE from three biological replicate assays, normalized to the *Actin* gene template quantity. Statistically significant differences between light and shading treatments are indicated (*t*‐test, *p < 0.05).

**FIGURE 3:**
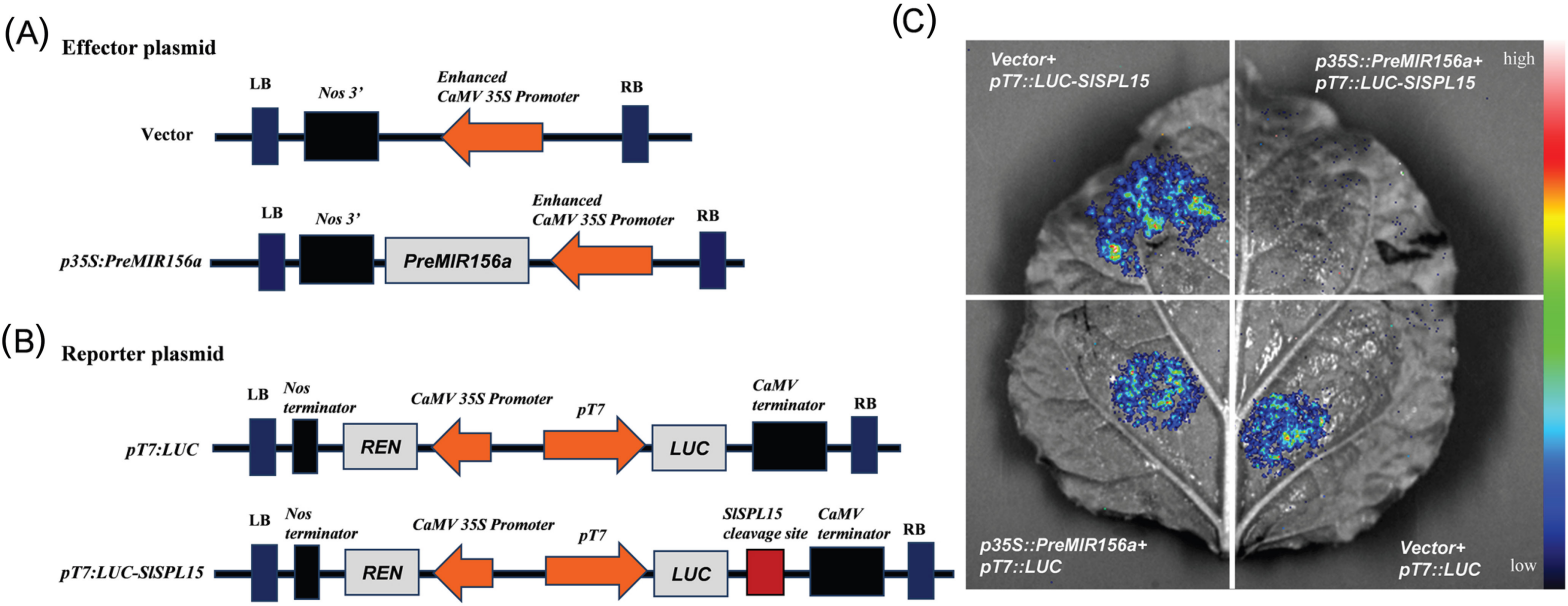
The effector and reporter plasmid schematic and Luciferase transient expression assay in *Nicotiana benthamiana* leaves. (A) Scheme of the effector plasmid. (B) Scheme of the reporter plasmid. (C) Different effector and reporter constructs were transformed into *Agrobacterium* GV3101 strain and infiltrated into tobacco leaves. Luminescence signals in the infiltrated area were detected 48 h post‐infiltration. The co‐expression of vector+*pT7:LUC*, vector+ *pT7:LUC‐SlSPL15*, and *p35S:PreMIR156a* + *pT7:LUC* were able to display luminescence signals, while the co‐expression of *p35S:PreMIR156a* + *pT7:LUC‐SlSPL15* did not show any luminescence signals.

Tomato, LA1996, which harbors the *Anthocyanin fruit* (*Aft*) gene, originated from a cross between *L. esculentum* × *L. chilense* (Georgiev 1972). When exposed to sunlight, the epidermis of the fruits accumulates pigments (Figure 2A). Additionally, the anthocyanin content in light‐treated fruits is significantly higher than that in fruits subjected to shading treatment (Figure 2B). qRT‐PCR analysis revealed that the transcription level of *SlSPL15* decreased during the light‐exposed MG stage of tomato fruits but significantly increased under shading conditions. Conversely, the expression trend of SlmiR156a is inversely correlated with that of *SlSPL15* (Figure 2C,D), suggesting a negative regulatory relationship between them. Furthermore, the SlmiR156 precursor was cloned from *Aft* tomato and its sequence contains the characteristic stem‐loop structure (Figure S1A). Sequencing analysis showed that SlmiR156a was primarily derived from the 5′ arm of the precursor (indicated by the red region; Figure S1B). A notable cleavage site at 3624 bp of *SlSPL15* was detected through degradome data analysis (Figure S1C). Furthermore, in transient expression assays via *Agrobacterium*‐mediated co‐infiltration of *35S:SlPreMIR156a* and *pT7:LUC‐SlSPL15* into tobacco leaves, the luciferase activity of LUC‐SlSPL15 was abolished due to SlmiR156a‐mediated degradation, resulting in the absence of detectable luminescence (Figure 3). These results demonstrate that SlmiR156a directly cleaves the 3′ terminus of SlSPL15‐associated LUC mRNA at its binding site through sequence‐specific targeting.

### 3.3 Over‐Expression of *SlSPL15* Displays Low‐Anthocyanin Accumulation in MG Fruits

**FIGURE 4:**
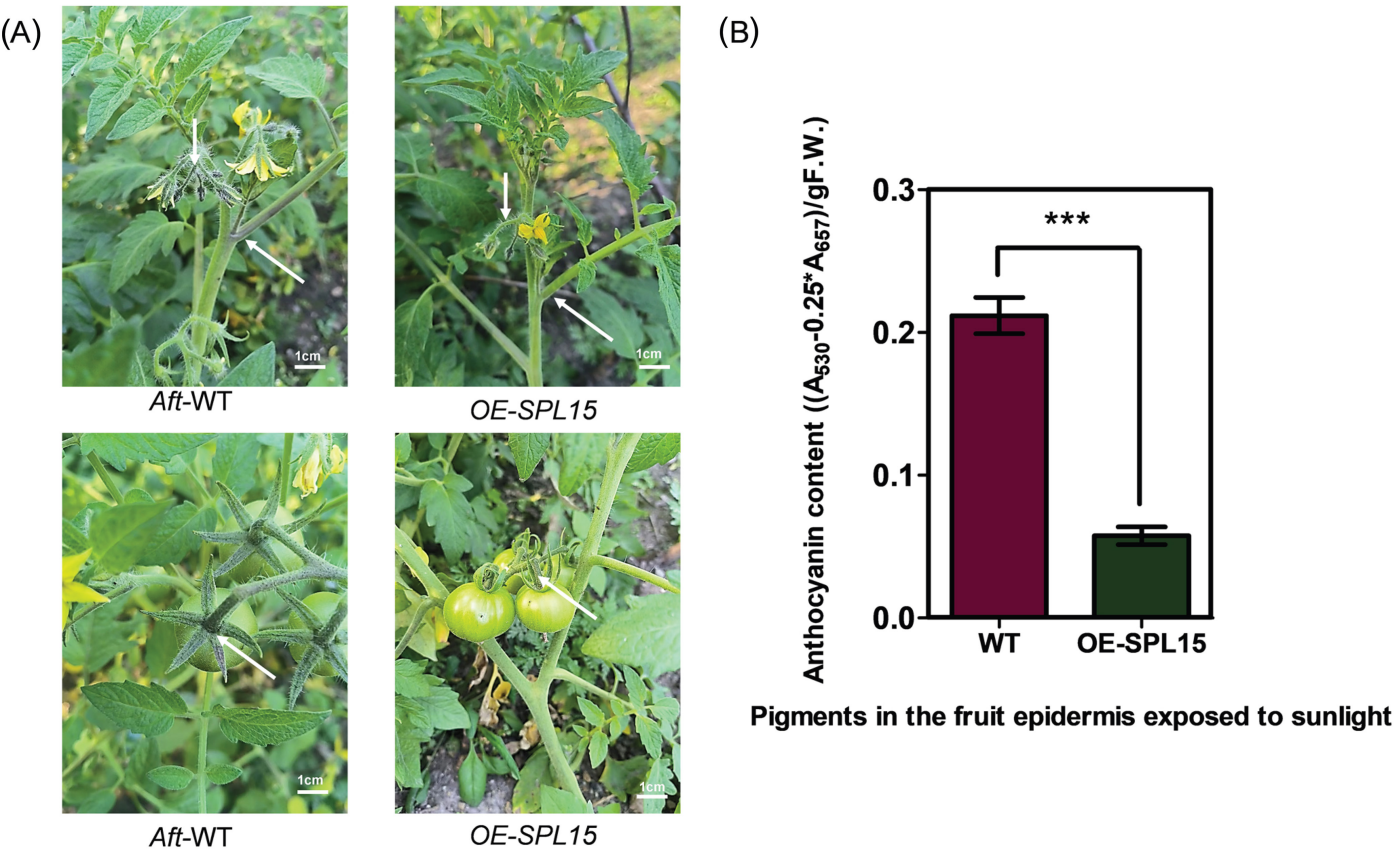
The identification, phenotype and anthocyanin content of *35S:SlSPL15* transformed *Aft* tomato plants. (A) Phenotype of *35S:SlSPL15* transformed *Aft* tomato plants (flowering stage and fruit stage). (B) Anthocyanin content of fruit epidermis (MG stage) in *Aft‐*WT plants and *OE‐SlSPL15* plants under sunlight. The arrows indicate the differences in pigment at the leaf base, nodes, and sepals between the wild type (*Aft*‐WT) and overexpression line (OE‐*SlSPL15*) in tomatoes.

To investigate the regulatory roles of the *SlSPL15* gene, overexpressed *SlSPL15* plants were obtained through *Agrobacterium*‐mediated transformation. The T0 transgenic tomato plants were grown in a greenhouse to generate seeds (T1), and the T1 seeds were subsequently screened on kanamycin‐supplemented media and confirmed by PCR using *KanR* (kanamycin resistance gene) primers. The *Aft* wild type (WT) displayed obvious pigment synthesis in the base of the leaves, nodes, sepals, and fruits. However, the overexpressed *SlSPL15* plants exhibited no significant pigment accumulation at the base and nodes of the leaves, nor in the fruits and sepals, as indicated by the arrows in Figure 4A. In MG fruits, the *Aft* WT exhibits a significantly greater anthocyanin content compared to the *OE‐SlSPL15* plants under sunlight. Low levels of anthocyanin synthesis were observed in the *OE‐SlSPL15* plants, confirming that the overexpression of the *SlSPL15* gene inhibits pigment accumulation in *Aft* tomato fruits (Figure 4B).

### 3.4 Differential Expression Analysis of Anthocyanin Biosynthetic Genes in Light‐Exposed and Shaded Fruits of *OE‐SlSPL15* Plants

**FIGURE 5:**
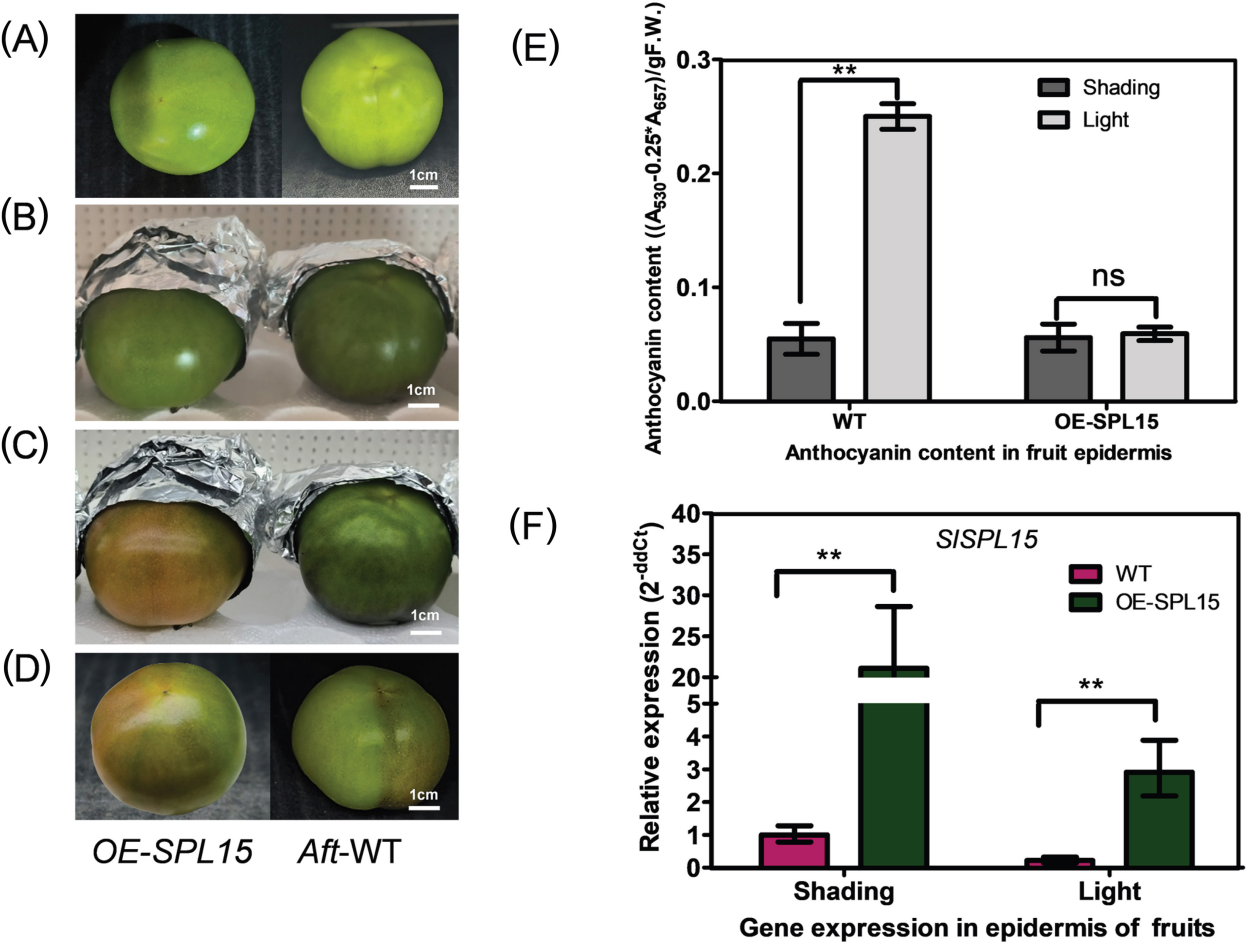
The light treatment, anthocyanin production detection and *SlSPL15* transcript abundance in the mature green (MG) development stage fruits of *Aft‐*WT plants and *OE‐SPL15* plants. (A) The MG stage fruits of *Aft‐*WT plants and *OE‐SPL15* plants. (B) The fruits were treated with one half exposed to LED light and the other half covered with aluminum foil for shading treatment. (C) The fruits were subjected to half exposure to light and half to shading for a duration of seven days. (D) The phenotype of anthocyanin biosynthesis in the fruits of *Aft‐*WT plants and *OE‐SPL15* with light and shading treatment. (E) The anthocyanin content of fruits of *Aft‐*WT plants and *OE‐SPL15* plants under light (half exposed to LED light) and shading (half covered with aluminum foil for shading). (F) The expression of *SlSPL15* in the mature green (MG) development stage fruits under light and shading treatment of *Aft*‐WT plants and *OE*‐*SPL15* plants.

**FIGURE 6:**
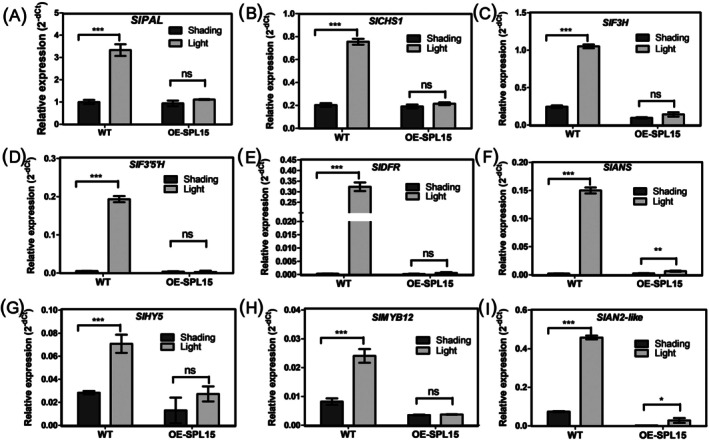
Expression profiles of anthocyanin biosynthesis‐related genes in mature green (MG) stage fruits of *Aft*‐WT and *OE*‐*SPL15* plants under light and shading treatment. (A–F) The relative expression of *SlPAL*, *SlCHS1*, *SlF3H*, *SlF3′5′H*, *SlDFR*, *SlANS* in MG stage fruits of *Aft*‐WT plants and *OE*‐*SPL15* plants under light (half exposed to LED light) and shading (half covered with aluminum foil for shading) treatment. (G–I) The relative expression of *SlHY5*, *SlMYB12*, and *SlAN2*‐*like* in MG stage fruits of *Aft*‐WT plants and *OE*‐*SPL15* plants under light (half exposed to LED light) and shading (half covered with aluminum foil for shading) treatment. The relative expression levels of the target gene were calculated using the 2^−ΔCt^ method. The ΔCt values were determined by subtracting the Ct value of the reference gene from the Ct value of the target gene for each sample.

To explore the molecular mechanism by which SlSPL15 negatively regulates light‐induced anthocyanin accumulation, we measured anthocyanin accumulation and spatiotemporal expression of biosynthetic pathway genes, including phenylalanine ammonialyase (*PAL*), chalcone synthase (*CHS1*), flavanone‐3‐hydroxylase (*F3H*), flavonoid 3′,5′‐hydroxylase (*F3*′*5*′*H*), and anthocyanidin synthetase (*ANS*) in fruits of *OE‐SlSPL15* plants under both light and shaded conditions. The study found that anthocyanin levels in *OE‐SlSPL15* plant fruits were reduced under light treatment compared to WT plants (Figure 5D). Moreover, there was almost no difference in pigment accumulation between light‐exposed and shaded fruits of *OE‐SlSPL15* plants (Figure 5E). Additionally, gene expression analysis showed the *OE‐SlSPL15* plants had a higher transcript level of *SlSPL15* in shaded and light‐exposed fruits compared to WT tomato (Figure 5F). Furthermore, the expression of anthocyanin biosynthetic genes *SlPAL*, *SlCHS1*, *SlF3*′*5*′*H*, *SlDFR*, and *SlF3H*, *SlANS* in light‐exposed fruits decreased to almost the same level as those in shaded fruits of *OE‐SlSPL15* plants. However, in WT plants, the transcription levels of these genes, which are involved in the biosynthesis of anthocyanins displayed an obvious light‐dependent expression trend (Figure 6A–F). Overall, the overexpression of *SlSPL15* affects genes related to anthocyanin biosynthesis, inhibiting light‐mediated anthocyanin accumulation in *Aft* tomato fruits.

### 3.5 The Transcription Level of Light Signal Transduction Factor SlHY5 and Light‐Responsive Regulatory Genes During the Anthocyanin Biosynthesis

Gene expression analysis suggests that the overexpression of *SlSPL15* may inhibit anthocyanin accumulation by disrupting light signal transduction and downregulating key regulatory genes in the anthocyanin biosynthetic pathway. Specifically, we investigated the expression of *SlHY5*, a central regulator of light‐induced anthocyanin synthesis, along with the expression of *SlAN2‐like* and *SlMYB12*. In *Pro35S:SlSPL15* plants, the expression levels of *SlHY5*, *SlMYB12*, and *SlAN2‐like* were significantly reduced in light‐exposed fruits (Figure 6G–I). This indicates that overexpressing *SlSPL15* adversely affects the transcription levels of regulatory genes, resulting in the decreased expression of anthocyanin biosynthetic genes in *Pro35S:SlSPL15* plants.

### 3.6 Nucleus‐Localized SlSPL15 Interacts With the Anthocyanin Biosynthesis Regulatory Protein SlAN2‐Like

**FIGURE 7:**
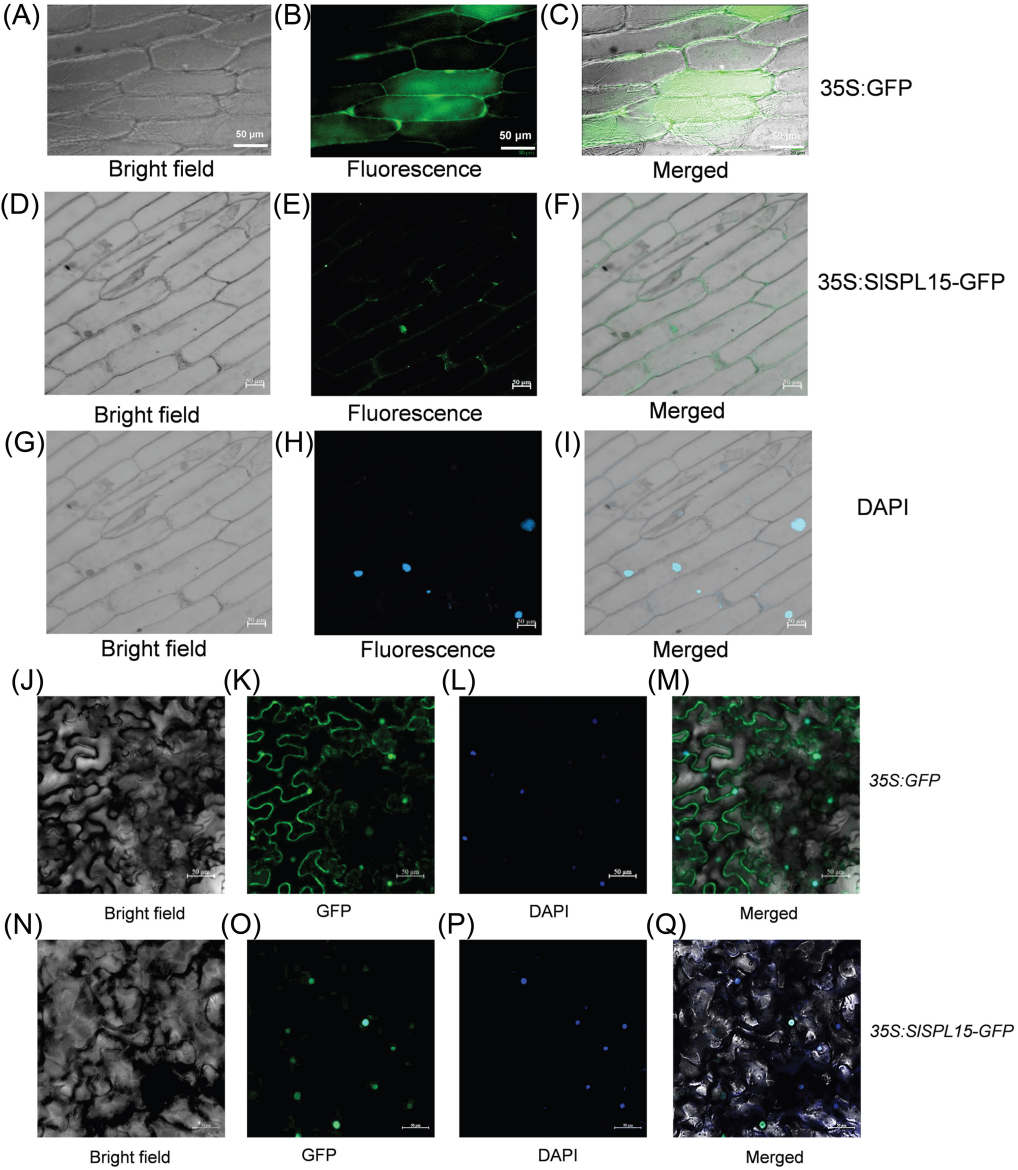
The subcellular localization analysis of SlSPL15 in onion epidermal and tobacco cells. (A) The subcellular localization of SlSPL15 in onion epidermal cells. (B) The subcellular localization of SlSPL15 in tobacco cells. Confocal fluorescent images of onion epidermal and tobacco cells expressing SlSPL15‐GFP fusion proteins captured using GFP, DAPI, and merged channels. GFP detection was performed with 488 nm excitation and emission observation at 505–550 nm, while DAPI was detected using 340 nm excitation and 488 nm emission. The fusion SlSPL15‐GFP was scanned by fluorescence field, bright field, and the subcellular localization was merged into onion epidermal and tobacco cells. All figures include scale bars that indicate a length of 50 μm.

To better elucidate the function of SlSPL15, we detected its subcellular localization by directly incorporating DNA into onion epidermal cells through vacuum infiltration. The results of fluorescence microscopy imaging showed that the *35S:SlSPL15‐GFP* fusion protein was localized in the nucleus (Figure 7A–I). Transient expression analysis of tobacco leaves infiltrated with *Agrobacterium* confirmed that the SlSPL15‐GFP fusion protein is also localized in the nucleus (Figure 7J–Q). The nuclear localization of SlSPL15 suggests that it may function as a transcription factor, directly interacting with gene promoters or associating with other regulatory proteins to modulate gene expression.

**FIGURE 8:**
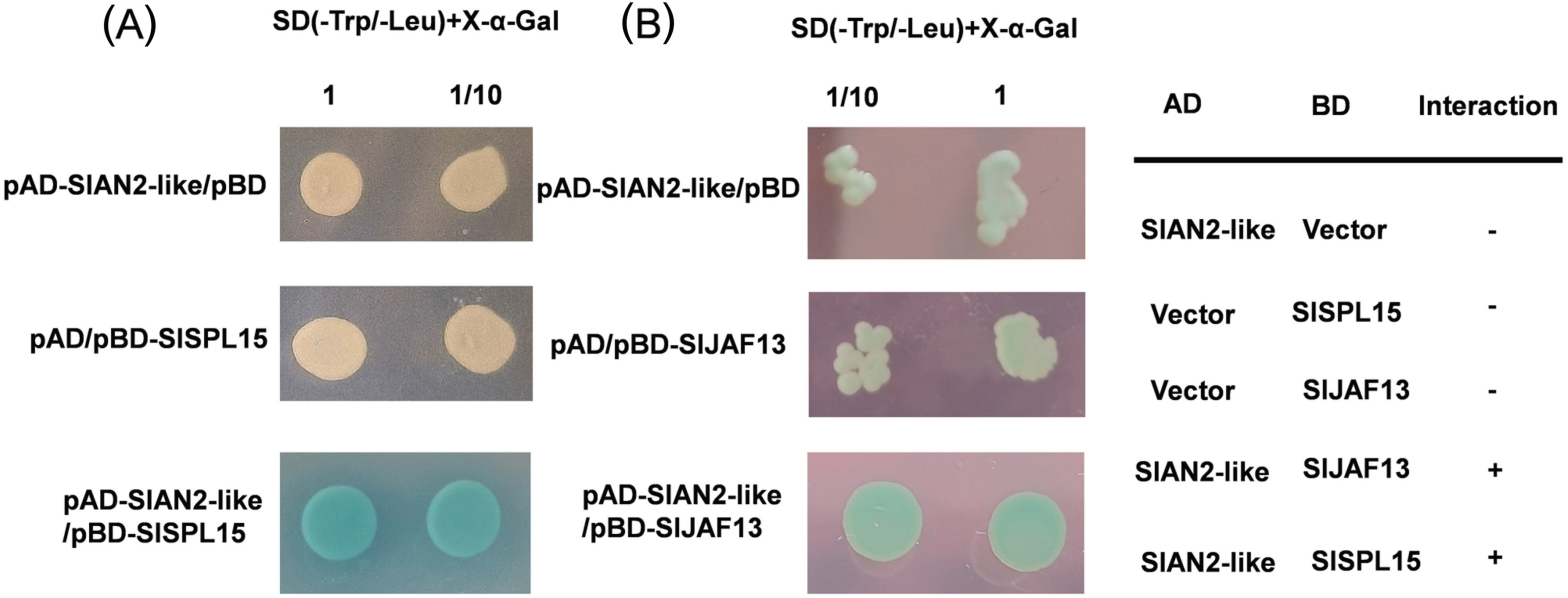
The interaction of SlSPL15/SlAN2‐like and SlJAF13/SlAN2‐like in yeast cells. (A) Yeast two‐hybrid assays confirmed direct binding between SlSPL15/SlAN2‐like transcription factors in vivo. (B) The detection of interaction between SlJAF13 and SlAN2‐like transcription factors in yeast cells. pBD‐SlSPL15 + pAD‐SlAN2‐like and pBD‐SlJAF13 + pAD‐SlAN2‐like constructs were co‐transformed into Y187 cells and grown on Minimal SD Agar Base media and Dropout Supplements without tryptophan and leucine, supplemented with 40 μg mL^−1^ X‐α‐gal. pBD‐SlSPL15 + pAD, pBD‐SlJAF13 + pAD, pAD‐SlAN2‐like + pBD were used as controls. 2 × 10^5^ cells were utilized for (1) and were subsequently diluted 10 times for (1/10). Images were captured after incubation at 28°C for 48 h.

The *Aft* phenotype in *Aft* tomatoes is attributed to the SlAN2‐like gene. SlAN2‐like has been revealed to act as an activator binding with the bHLH regulator SlJAF13 to form the MYB‐bHLH‐WD40 complexes regulating anthocyanin accumulation (Yan et al. 2020). In *Arabidopsis*, SPL9 disrupts the stabilization of the MYB‐bHLH‐WD40 complex by interacting with PAP1 and MYB113, thereby inhibiting the expression of anthocyanin biosynthetic genes (Gou et al. 2011). The overexpression of *SlSPL15* in *Aft* tomato plants indicates that SlSPL15 downregulates the transcriptional levels of anthocyanin biosynthetic genes and pigment accumulation. In addition, the expression of transcription factors SlHY5, SlMYB12, and SlAN2‐like was also negatively regulated by SlSPL15. We propose that SlSPL15 competes with SlJAF13 for binding to SlAN2‐like, influencing the formation of the MBW complex. We employed a yeast two‐hybrid assay to examine the interaction between SlSPL15 and SlAN2‐like. Consistent with the previous report (Yan et al. 2020), SlAN2‐like could directly interact with SlJAF13. Moreover, the assay also demonstrated that SlSPL15 directly binds to SlAN2‐like (Figure 8).

## 4 Discussion

### 4.1 The *SlSPL15* Gene Targeted by SlmiR156a Is a Potential Regulator in the Anthocyanin Biosynthetic Pathway in Tomato Plants

The evolutionarily conserved miR156/SPL regulatory module serves as a key regulator in plant growth development (Wang and Wang 2015), signal transduction (Jadhao et al. 2023), anthocyanin accumulation (J. Gou et al. 2011; Wang et al. 2020), and plant defense (Feyissa et al. 2021). Previous studies have demonstrated that SlmiR156 modulates ovary and fruit development (Ferreira e Silva et al. 2014), fruit ripening and softening (Chen et al. 2015), inflorescence morphogenesis (Cui et al. 2020), shoot branching (Barrera‐Rojas et al. 2023), fruit size, and yield (Zhang, Zou, et al. 2011) in tomato. SPL proteins are plant‐specific TFs defined by their characteristic SBP motif, which are integral to regulating plant growth and development (Mehtab et al. 2024). MiR156 mediates cleavage of *SPL* mRNAs by binding to a conserved MRE (miRNA‐responsive element) located in either the coding sequence or 3′ UTR (3′ untranslated regions) (Wang and Wang 2015). In our study, the 17 *SPL* genes identified in tomato correspond to the number of orthologous *SPL* genes in *Arabidopsis*. These *SPL* genes, containing between 1 and 12 exons, indicate a complex evolutionary process that has contributed to their diverse functions. Moreover, we identified four segmental duplication events associated with seven *SlSPL* genes across six chromosomes (Figure 1A). The calculated Ka/Ks substitution rate for these pairs of gene duplications suggests that strong purifying selection has acted to eliminate deleterious mutations, thereby preserving the gene family's essential functions. Phylogenetic analysis classifies the *SPL* gene families in tomato and *Arabidopsis* into six groups, suggesting that different SPL orthologs may perform analogous functions in these species. Specifically, SlSPL15, AtSPL15, and AtSPL9 cluster together in Group V, indicating potential functional similarities. Functional analysis revealed that *AtSPL9* impairs anthocyanin biosynthesis by disrupting the MBW complex (Gou et al. 2011), implying an evolutionarily conserved role for SlSPL15 in tomatoes.

### 4.2 SlmiR156a Targeted *SlSPL15* for Cleavage and Their Transcription Level Is Related to the Anthocyanin Content in Light‐Exposed and Shading Fruits of *Aft* Tomatoes

Light serves as a key environmental signal that significantly influences plant growth and development. Light signaling transduction factors, like PIF3, CYR1, UVR8, and HY5, play crucial roles in anthocyanin biosynthesis across various light conditions (Favory et al. 2009; Liu et al. 2015, 2018; Shin et al. 2007). In particular, HY5 is known to activate various light response pathways regulated by the combined action of phytochromes, cryptochromes, and UVR8 photoreceptors, establishing it as a central regulator of anthocyanin biosynthesis (Liu et al. 2023). In *Arabidopsis*, HY5 physically interacts with the miR156 promoter region to modulate its transcriptional activity (Zhang, He, et al. 2011). In *Aft* tomato fruits exposed to light, there is an increase in anthocyanin accumulation, which correlates with an elevated transcription level of SlmiR156a and a decreased expression of *SlSPL15*. This suggests a negative regulatory relationship between the expression of SlmiR156a and *SlSPL15* under conditions of light induction and shading. Dual‐luciferase reporter assays further confirmed that SlmiR156a mediates post‐transcriptional regulation of *SlSPL15* through sequence‐specific mRNA cleavage (Figure 3). Notably, a functionally conserved miR156a/SPL9 regulatory module has also been identified in red pear (*Pyrus* L.), mediating light‐responsive anthocyanin biosynthesis (Liu et al. 2021). We postulate that the SlmiR156a/SlSPL15 module may similarly regulate anthocyanin biosynthesis in *Aft* tomato fruits.

### 4.3 SlSPL15 Acts as a Negative Regulator Involved in Multi‐Signal Transduction and Regulatory Pathways Affecting Light‐Induced Anthocyanin Biosynthesis in Tomato Fruits

The SPL transcription factors have been well characterized across a wide range of plant species, including *Arabidopsis* (Cardon et al. 1999; Chao et al. 2017; Jung et al. 2012, 2016; Xing et al. 2013), apple (Li et al. 2023; Li et al. 2013; Ma, Xue, et al. 2021), grapevine (Cui et al. 2018; Su et al. 2021), tomato (Salinas et al. 2012), Litchi (Xu et al. 2024), blueberry (Feng, Zhou, et al. 2023), cotton (Cai et al. 2018; Zhang et al. 2015), and wheat (Zhu et al. 2020) where they perform multiple functions. Additionally, SPL9 in *Arabidopsis* (Gou et al. 2011) and pear (Liu et al. 2021), as well as SPL12 in blueberry (Li et al. 2020), have been validated to participate in anthocyanin biosynthesis. Moreover, the miR156‐SPL module is also known to fine‐tune the anthocyanin biosynthetic pathway in poplar (Wang et al. 2020). In the present study, we observed reduced anthocyanin accumulation in MG‐stage developing fruits of the *OE‐SlSPL15 Aft* tomato line compared with WT fruits. This reduction was accompanied by a decline in the transcription levels of both regulatory genes, namely *SlHY5*, *SlMYB12*, and *SlAN2‐like*, and structural genes such as *SlPAL*, *SlCHS1*, *SlF3H*, *SlDFR*, *SlF3′5′H*, and *SlANS*. Moreover, in the fruits of the *OE‐SlSPL15 Aft* tomato line, no significant variation was observed in the transcription levels of these anthocyanin biosynthetic genes between light‐induced and light‐shading conditions (Figure 6A–I). In *OE‐SlSPL15* tomato fruits, the expression of *SlSPL15* also differed significantly between light‐induced and shading‐treated samples (Figure 5). This suggests that *SlSPL15* may be post‐transcriptionally regulated by light‐induced SlmiR156a. These findings indicate that SlSPL15 plays a negative regulatory role in anthocyanin biosynthesis. Our results also demonstrate that the upregulated expression of *SlSPL15* in the *Aft* tomato exerts a negative impact on the expression of genes associated with the anthocyanin synthesis pathway, thereby consequently influencing anthocyanin accumulation.

As a transcription factor, SlSPL15 is proposed to negatively regulate anthocyanin synthesis through two primary pathways. First, SlSPL15 can form a heterodimer with the MYB protein SlAN2‐like, potentially disrupting the MBW regulatory complex (comprising SlAN2‐like/SlJAF13/SlAN11 and SlAN2‐like/SlAN1/SlAN11) which leads to transcriptional repression of anthocyanin biosynthetic genes and, consequently, a reduction in pigment accumulation. Second, SlSPL15 might serve as a negative transcriptional regulator by directly interacting with GTAC *cis*‐elements in the promoter regions of structural or regulatory genes, thereby modulating their expression and affecting anthocyanin accumulation. Functional analysis uncovered that SlSPL15 physically interacts with SlAN2‐like (Figure 8). This interaction suggests that accumulated SlSPL15 forms heterodimers with SlAN2‐like, thereby impairing MYB‐dependent transcriptional activation of anthocyanin biosynthesis genes. A previous study revealed that SlHY5 directly targets the ACE‐box motif in the *SlAN2*‐*like* promoter for light‐responsive activation (Sun et al. 2020) and regulates the expression of anthocyanin biosynthetic genes (*CHS1*, *CHS2*, *CHI*, *F3H*, *F3′H*, *DFR*, *ANS*, and *3‐GT*) (Wang, Wang, et al. 2021). Subsequently, *SlAN2‐like* transcripts were significantly induced in *Aft* fruit peels under light treatment. However, elevated levels of SlSPL15 disrupt the MBW transcriptional complex through physical interaction with SlAN2‐like, thereby repressing transcription of anthocyanin biosynthetic genes in *OE‐SlSPL15 Aft* tomato fruits, despite the critical role of SlHY5 in light‐induced anthocyanin accumulation. Moreover, the overexpression of *SlSPL15* leads to the transcriptional downregulation of anthocyanin biosynthetic genes, presumably by acting as a negative transcription factor that targets the promoters of these genes. Similar mechanisms have been observed with other SPL transcription factors. For instance, SPL9 in *Arabidopsis* binds to the GTAC boxes in the *DFR* promoter (Gou et al. 2011), while SmSPL7 in *Salvia miltiorrhiza* inhibits the expression of *SmTAT1* and *Sm4CL9* by binding to their promoters (Chen et al. 2021). In wild apples ( *Malus sieversii* ), MsSPL13 represses *MsYUCCA3/5* and *MsPIN7* expression (Feng, Zhang, et al. 2023), suggesting a common regulatory role for SPL proteins in modulating gene expression. These examples provide a framework for understanding how SlSPL15 might exert its effects on anthocyanin biosynthesis through the downregulation of key regulatory genes in *Aft* tomato fruits.

Our study demonstrated that in *OE‐SlSPL15 Aft* tomato fruits, the expression of *SlHY5* and *SlAN2‐like* is suppressed. The results suggest that SlSPL15 might function as a negative regulator by suppressing the transcription of *SlHY5* and *SlAN2‐like*, or either as a positive regulator by activating genes that negatively regulate anthocyanin synthesis. The indirect suppression of *SlHY5* or *SlAN2‐like* influences the expression of genes involved in anthocyanin synthesis, leading to reduced anthocyanin production. SlSPL15 may be involved in various signaling and regulatory pathways related to light‐induced anthocyanin accumulation in *Aft* tomato fruits. Nonetheless, further investigation is needed to elucidate the detailed molecular mechanisms.

### 4.4 A Model Illustrating SlSPL15's Role in Regulating Anthocyanin Biosynthesis

**FIGURE 9:**
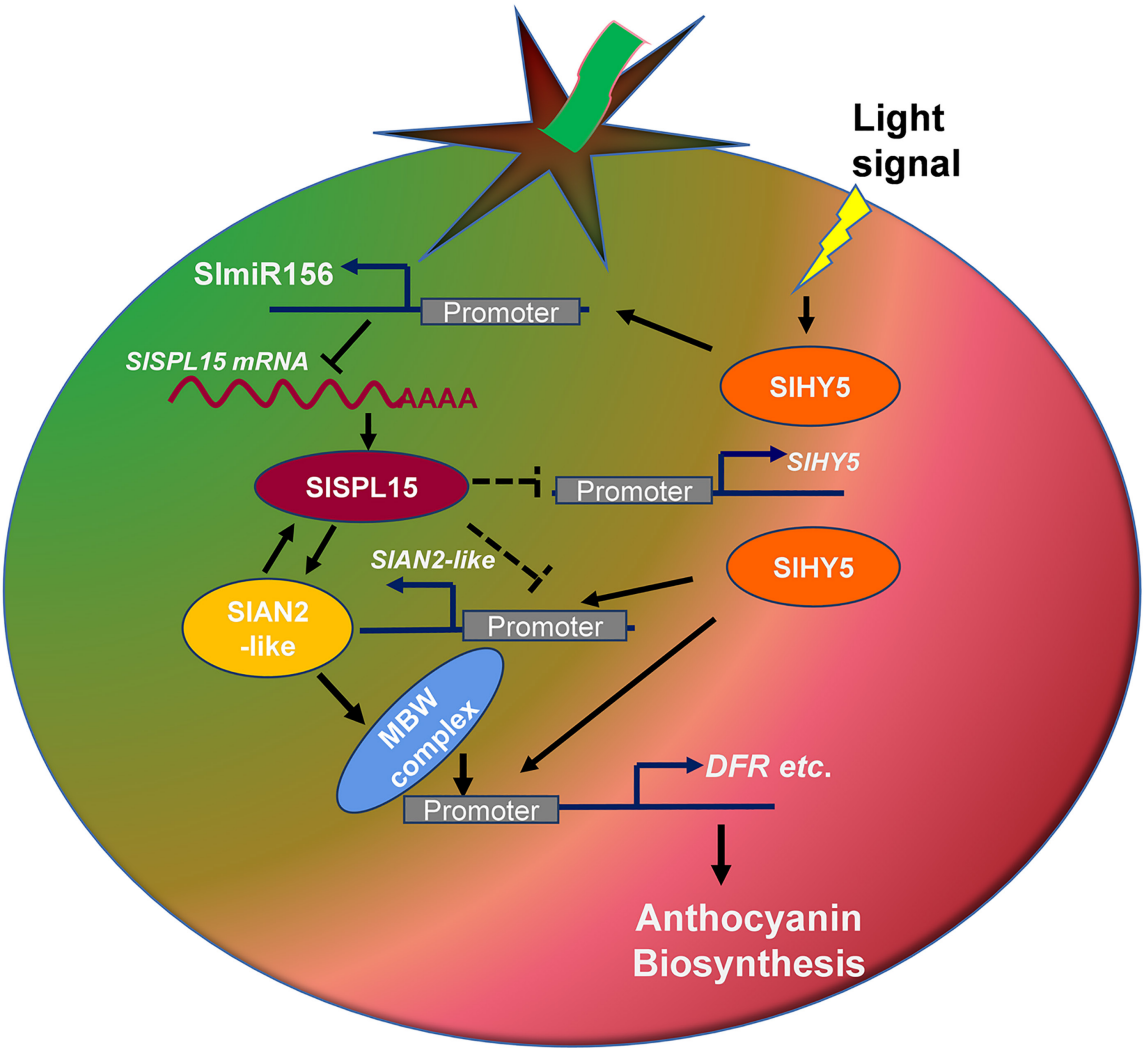
A regulatory model of *SlSPL15* negatively influencing SlmiR156a‐mediated light‐induced anthocyanin biosynthesis in *Aft* tomatoes. In the light, SlHY5 positively regulates the MBW complex and anthocyanin biosynthetic genes, meanwhile SlHY5 actives the expression of SlmiR156a, which results in the cleavage of *SlSPL15* mRNA and a subsequent decrease in SlSPL15 protein levels in the complex. The anthocyanin accumulates in the *Aft* fruits of tomato under light‐exposed. → means positive regulation or interaction, while 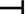 means negative regulation or interaction. The dark blue arrows indicate the transcription activation of the regulated target genes. The dotted lines in the figure represent inferred possible regulatory mechanisms that have not been experimentally validated.

Our study presents a model illustrating the regulatory role of the SlmiR156a/SlSPL15 module in the anthocyanin biosynthesis pathway of *Aft* tomato fruits (Figure 9). In light‐exposed fruits, SlHY5 is activated and modulates light‐induced expression of genes such as SlmiR156a, *SlAN2*‐*like*, and genes involved in anthocyanin biosynthesis. SlmiR156a functions as a negative regulator of *SlSPL15* which in turn reduces the inhibition of SlAN2‐like, thereby reducing interference with the MBW complex. The increased activity of these regulatory elements enhances the expression of biosynthetic genes in the anthocyanin pathway, resulting in higher pigment production.

## 5 Conclusions

This research explores the role of *SlSPL15* in the anthocyanin biosynthesis of *Aft* tomato fruits. Our findings indicate that *SlSPL15* is regulated by SlmiR156a through cleavage and functions as a suppressor of anthocyanin biosynthesis under light‐shading conditions. The interaction between SlSPL15 and the SlAN2‐like protein forms a heterodimer that suppresses the expression of structural genes responsible for anthocyanin biosynthesis, leading to reduced anthocyanin accumulation. Overexpression of *SlSPL15* also results in decreased expression of the light signal transduction factor SlHY5 and the anthocyanin biosynthetic gene *SlAN2‐like*, which negatively impacts the anthocyanin biosynthesis pathway. These findings enhance our understanding of the SlmiR156a‐SlSPL15 module's function in the light‐regulated network of anthocyanin accumulation in *Aft* tomato.

## Author Contributions

C.X., S.Q., F.G., and H.W. constructed the vectors and finished the experiment of splice site detection, yeast two hybridization, and genetic transformation assay. J.L. and J.Z.L. detected the subcellular location of SlSPL15 and performed qRT‐PCR. B.Z. designed the work, wrote the manuscript, and analyzed the data from sequencing. W.W. took part in the design of the work, the interpretation of data for the work, and the reviewing and editing of the manuscript. All authors have read and agreed to the published version of the manuscript.

## Conflicts of Interest

The authors declare no conflicts of interest.

## Supporting information


**Data S1:** ppl70471‐sup‐0001‐supinfo.docx.

## Data Availability

The data underlying this article are available in the article and in Supporting Information.
