# Supplementary material for: SlSPL15 : A Negative Regulator Targeted by SlmiR156a Participates in Regulating the Light‐Induced Anthocyanin Biosynthesis of Aft Tomato Fruits
Source: Physiol Plant. 2025 Aug 30;177(5):e70471. doi: 10.1111/ppl.70471 (PMC12397961; doi:10.1111/ppl.70471)
Supplement: Supplementary file 1 — Data S1: ppl70471‐sup‐0001‐supinfo.docx. [file PPL-177-e70471-s001.docx]

**SlSPL15: A negative regulator targeted by SlmiR156a participates in regulating the light-induced anthocyanin biosynthesis of *Aft* tomato fruits**

**Chuyao Xu ^1, ‡^, Siyue Qi ^1, ‡^, Fuchang Guo ^1^, Hui Wang ^1^, Ji Li ^1^, Jiazhen Li ^1^, Weilin Wu ^2,^ *, Bo Zhou ^1,^ ***

^1^ College of Life Science, Northeast Forestry University, Harbin 150040, China.

^2^ Agricultural College, Yanbian University, Yanji 133002, China.

^‡^ These authors contributed equally to this work

**Correspondence:**

*Corresponding authors,

E-mails: [zhoubo@nefu.edu.cn](mailto:zhoubo@nefu.edu.cn), wlwu@ybu.edu.cn

Edited by: Sheng-Hong Li


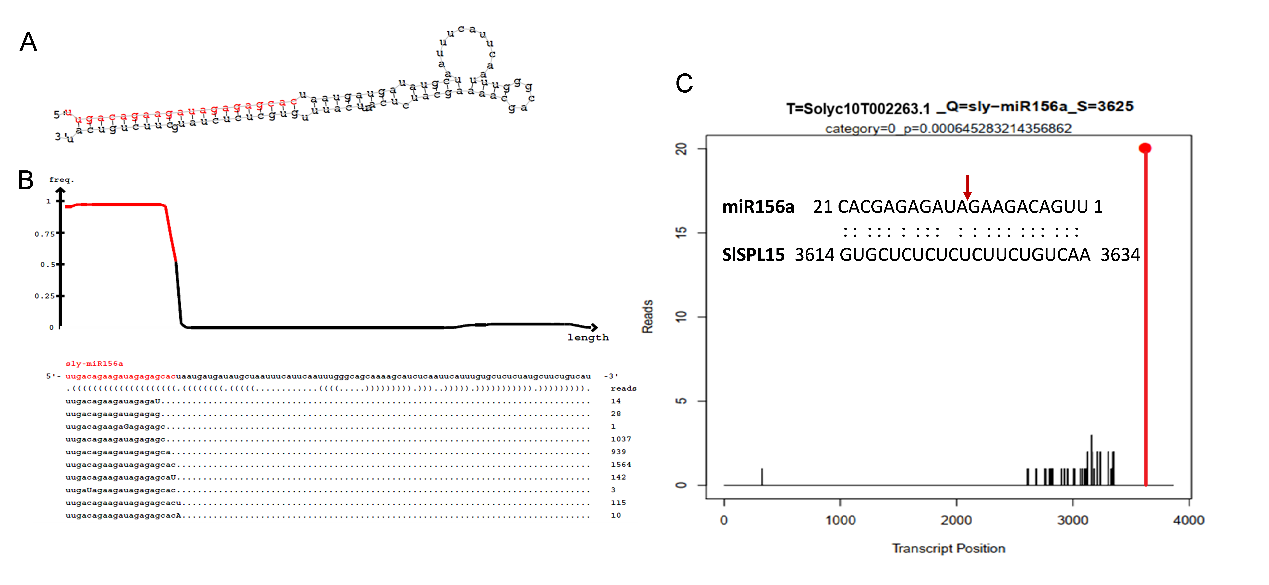


**Fig. S1 The miRNA hairpin structure and mature miR156a binding site for cleavage of *SlSPL15***

(A) The miRNA hairpin structure of miR156a precursor, red region is mature SlmiR156a. (B) The sequenced mature miR156a in *Aft* tomato. A line diagram illustrating the mapping of clean reads from small RNA sequencing to the genome-based prediction of stem-loop structure sequences. (C) The cleavage site of *SlSPL15* targeted by SlmiR156a. Cleavage sites inferred from degradome data are indicated, with red arrows denoting the strongest signals.

| **Table S1 The primer sequences used for qRT-PCR assay and vector construction** | | | | |
| --- | --- | --- | --- | --- |
| **Primer used** | **Name** | | **Primer sequences (5' to 3')** | |
|  | *SlDFR* | AGCTTAATCACTGCCCTTTCAC | | TCGCACCATCTTAGCCACAT |
|  | *SlMYB12* | ATGCCGGATTATTGAGATGCG | | GCTATCAACTTTTCGACTTAGATGAGAG |
|  | *SlAN2-like* | ATGGTCACTTATTGCTGGGAGA | | TTCACTTGAGAAGGTTCGAGGT |
|  | *SlHY5* | AGCGACGAGTTCTATTGCC | | CCTTGCTTGTTGTGCTGATACT |
|  | *SlPAL* | GGAATTGCAGGGTTGCCACTTT | | AAGGCCGCGTTGCCTAAAGAAG |
|  | *SlCHS1* | TGGTCACCGTGGAGGAGTATC | | GATCGTAGCTGGACCCTCTGC |
|  | *SPL15* | CCAACAGTGTAGCAGGTTCCA | | GGTGTGAGCTGAAGTCTACCA |
| **qRT-PCR** | *SlF3H* | GTGAAAAGTTGATGGATTTGGC | | GTAATGGTTCCTGGATCGGTGTGT |
|  | *SlF3'5'H* | GGTACATGTGGGATGGTTGTTGC | | ACTTCCAACGTGGTCCATAGGG |
|  | *SlANS* | GAACTAGCACTTGGCGTCGAA | | TTGCAAGCCAGGCACCATA |
|  | *PremiR156* | TATAATTGAAATTGATAGGAAGA | | TATTTTTACGAAAGAGGTGAAGG |
|  | *Actin* | GGGATGGAGAAGTTTGGTGGTGG | | CTTCGACCAAGGGATGGTGTAGC |
| **Gene clone** | *SlSPL15* | CACCAGTATCTTGTTTTTCC | | CATCTAAACGAAGGGGAG |
|  | *SlAN2-like* | ATGAATATTGCCAAGACATTGG | | CTAATTAAATAGATTCCATAGG |
|  | *SlpremiR156a* | TATAATTGAAATTGATAGGAAGA | | TATTTTTACGAAAGAGGTGAAGG |
| **Y2H Vector construction** | *SlAN2-like-pAD* | aagagatcgaattaggatccATGAATATTGCCAAGACATTGG | | actcactatagggctctagaCTAATTAAATAGATTCCATAGG |
|  | *SlJAF13-pBD* | ggcctcgagcccgggtcgacTCATGGCTATGGGACACCAA | | tatagggctctagagtcgacAGATTTCCATACTACTCTCTG |
|  | *SlSPL15-pBD* | ggcctcgagcccgggtcgacTCATGGAACTGGGTTCAGTGTCTTC | | tatagggctctagagtcgacAAGAGTCCAGTGCACATTCTG |
| ***SlSPL15* overexpression vector** | *SLSPL15_pBI121* | cacgggggactctagaGGATCCATGGAACTGGGTTCAGTGTCTT | | agggactgaccacccggGGATCCAAGAGTCCAGTGCACATTCTG |
| ***SlpremiR156a* overexpression vector** | *SlpremiR156a-pA7-GFP* | ccgctcgagTATAATTGAAATTGATAGGA | | cgggatccTATTTTTACGAAAGAGGTGAA |
| ***pGreenII 0800-pT7-LUC*** | *pT7-LUC primer* | TAATACGACTCACTATAGGTCGACGGTATCGATAAGCTTGATCCACTAGTTCTAGAGCG | | AAGCTTATCGATACCGTCGACCTATAGTGAGTCGTATTACCAGAGTTCTTACCTTGAAGC |
| ***pT7-LUC-MRE vector*** | *pT7-LUC-MRE primer* | CTCGAGGCACCAGAGTGCTCTCTCTCTTCTGTCAAGGTACCTTCTAGAGAATTCGGTACGC | | GGTACCTCTGGTGCCTCGAGGTGCTCTCTCTCTTCTGTCAATTACACGGCGATCTTTCCGC |
| **Transformed plants identification** | *kanamycin resistance gene* | GAATCGGGAGCGGCGATACC | | CCACCAAGCGAAACATCGCAT |

| **Table S2 The Ka/Ks ratios of duplicated SlSPL gene pairs in *Aft* tomato** | | | | | | | |  |  |  |  |  |
| --- | --- | --- | --- | --- | --- | --- | --- | --- | --- | --- | --- | --- |
| Seq_1 | Seq_2 | Ka | Ks | Ka_Ks | EffectiveLen | AverageS-sites | AverageN-sites | cN | cS | pN | pS | Note |
| Solyc02T001535.2 | Solyc07T002101.1 | 0.560921 | NaN | NaN | 444 | 98.83333 | 345.1667 | 136.3333 | 75.66667 | 0.394978 | 0.765599 | High Sequence Divergence Value (pS>=0.75) |
| Solyc07T002101.1 | Solyc07T002480.1 | 0.391034 | 2.975254 | 0.131429 | 318 | 67.5 | 250.5 | 76.33333 | 49.66667 | 0.304724 | 0.735802 |  |
| Solyc01T002662.1 | Solyc10T000744.1 | 0.21048 | 1.192843 | 0.176453 | 810 | 179.75 | 630.25 | 115.6667 | 107.3333 | 0.183525 | 0.597126 |  |
| Solyc03T002758.1 | Solyc05T000648.1 | 0.510814 | 2.129686 | 0.239854 | 1407 | 315.0833 | 1091.917 | 404.5 | 222.5 | 0.37045 | 0.706162 |  |

| **Table S3 The predicted cleavage sites of SlSPL genes targeted by SlmiR156a** | | | | | | | | |  |  |  |  |  |  |
| --- | --- | --- | --- | --- | --- | --- | --- | --- | --- | --- | --- | --- | --- | --- |
| miRNA_Acc. | Target_Acc.(V5) | Target_Acc.(V2.4) | SPL family member | Expectation | UPE$ | miRNA_start | miRNA_end | Target_start | Target_end | miRNA_aligned_fragment | alignment | Target_aligned_fragment | Inhibition | Multiplicity |
| sly-miR156a | Solyc02T001535.2 | Solyc02g077920 | SlSPL17 | 0 | -1 | 1 | 21 | 21 | 41 | UUGACAGAAGAUAGAGAGCAC | :::::::::::::::::::: | AUGCUCUCUAUCUUCUGUCAA | Cleavage | 1 |
| sly-miR156a | Solyc04T001200.1 | Solyc04g045560 | SlSPL5 | 1.5 | -1 | 1 | 21 | 21 | 41 | UUGACAGAAGAUAGAGAGCAC | ::::::::: ::::::::::: | GUGCUCUCUCUCUUCUGUCAA | Cleavage | 1 |
| sly-miR156a | Solyc10T002263.1 | Solyc10g078700 | SlSPL15 | 1.5 | -1 | 1 | 21 | 21 | 41 | UUGACAGAAGAUAGAGAGCAC | ::::::::: ::::::::::: | GUGCUCUCUCUCUUCUGUCAA | Cleavage | 1 |
| sly-miR156a | Solyc05T001030.1 | Solyc05g015510 | SlSPL8 | 1.5 | -1 | 1 | 21 | 21 | 41 | UUGACAGAAGAUAGAGAGCAC | ::::::::: ::::::::::: | GUGCUCUCUCUCUUCUGUCAA | Cleavage | 1 |
| sly-miR156a | Solyc05g012040.2.1 | Solyc05g012040 | SlSPL7 | 2 | -1 | 1 | 21 | 21 | 41 | UUGACAGAAGAUAGAGAGCAC | ::::::::: ::::::::::. | GUGCUCUCUCUCUUCUGUCAG | Cleavage | 1 |
| sly-miR156a | Solyc12T001656.2 | Solyc12g038520 | SlSPL16 | 2.5 | -1 | 1 | 21 | 21 | 41 | UUGACAGAAGAUAGAGAGCAC | ::::::::: :::::::::: | GUGCUCUCUCUCUUCUGUCAU | Cleavage | 1 |
| sly-miR156a | Solyc05T001068.1 | Solyc05g015840 | SlSPL9 | 2.5 | -1 | 1 | 21 | 21 | 41 | UUGACAGAAGAUAGAGAGCAC | ::::::::: :::::::::: | GUGCUCUCUCUCUUCUGUCAU | Cleavage | 1 |
| sly-miR156a | Solyc07T002101.1 | Solyc07g053810 | SlSPL11 | 2.5 | -1 | 1 | 21 | 21 | 41 | UUGACAGAAGAUAGAGAGCAC | :::::::: :::::::::: | UUGCUCUCUCUCUUCUGUCAU | Cleavage | 1 |
| sly-miR156a | Solyc03T002758.1 | Solyc03g114850 | SlSPL14 | 2.5 | -1 | 1 | 21 | 21 | 41 | UUGACAGAAGAUAGAGAGCAC | :::::::: :::::::::: | AUGCUCUCUCUCUUCUGUCAU | Cleavage | 1 |
| sly-miR156a | Solyc10T000422.1 | Solyc10g009080 | SlSPL13 | 2.5 | -1 | 1 | 21 | 21 | 41 | UUGACAGAAGAUAGAGAGCAC | ::::::: :::::::::: | ACGCUCUCUCUCUUCUGUCAU | Cleavage | 1 |
| sly-miR156a | Solyc07T002480.1 | Solyc07g062980 | SlSPL12 | 3.5 | -1 | 1 | 21 | 21 | 41 | UUGACAGAAGAUAGAGAGCAC | ::::. :: ::::::::::. | AUGCUUACUCUCUUCUGUCAG | Cleavage | 1 |
